# Comprehensive analysis of IgA nephropathy expression profiles: identification of potential biomarkers and therapeutic agents

**DOI:** 10.1186/s12882-021-02356-4

**Published:** 2021-04-19

**Authors:** Alieh Gholaminejad, Yousof Gheisari, Sedigheh Jalali, Amir Roointan

**Affiliations:** 1 Regenerative medicine research center, Isfahan University of Medical Sciences, Isfahan, Iran; 2 Department of Pediatrics, The University of Melbourne, Melbourne, Australia

**Keywords:** IgA nephropathy, Computational biology, Gene expression, Gene regulatory network, Biomarkers

## Abstract

**Background:**

IgA nephropathy (IgAN) is a kidney disease recognized by the presence of IgA antibody depositions in kidneys. The underlying mechanisms of this complicated disease are remained to be explored and still, there is an urgent need for the discovery of noninvasive biomarkers for its diagnosis. In this investigation, an integrative approach was applied to mRNA and miRNA expression profiles in PBMCs to discover a gene signature and novel potential targets/biomarkers in IgAN.

**Methods:**

Datasets were selected from gene expression omnibus database. After quality control checking, two datasets were analyzed by Limma to identify differentially expressed genes/miRNAs (DEGs and DEmiRs). Following identification of DEmiR-target genes and data integration, intersecting mRNAs were subjected to different bioinformatic analyses. The intersecting mRNAs, DEmiRs, related transcription factors (from TRRUST database), and long-non coding RNAs (from LncTarD database) were used for the construction of a multilayer regulatory network via Cytoscape.

**Result:**

“GSE25590” (miRNA) and “GSE73953” (mRNA) datasets were analyzed and after integration, 628 intersecting mRNAs were identified. The mRNAs were mainly associated with “Innate immune system”, “Apoptosis”, as well as “NGF signaling” pathways. A multilayer regulatory network was constructed and several hub-DEGs (Tp53, STAT3, Jun, etc.), DEmiRs (miR-124, let-7b, etc.), TFs (NF-kB, etc.), and lncRNAs (HOTAIR, etc.) were introduced as potential factors in the pathogenesis of IgAN.

**Conclusion:**

Integration of two different expression datasets and construction of a multilayer regulatory network not only provided a deeper insight into the pathogenesis of IgAN, but also introduced several key molecules as potential therapeutic target/non-invasive biomarkers.

## Highlights

- Innate immune system, apoptosis, and NGF signaling were the most linked pathways to the pathogenesis of IgAN.
- A multilayer regulatory network was constructed using 3 regulatory elements: long non-coding RNAs, differentially expressed microRNAs and transcription factors.
- Tp53, STAT3, and Jun were among top DEGs in the constructed multilayer regulatory network.
- miR-124 was introduced as a non-invasive biomarker for detection of IgAN disease.

## Background

IgA nephropathy (IgAN) is known as the most common primary glomerular disorder and one of the main causes of kidney failure worldwide [1–3]. Up to 40% of IgAN patients will finally progress to end-stage renal disease (ESRD), a condition with huge stress for the patients and a massive economic burden for the governments [4]. Besides hematuria and proteinuria, renal biopsy for checking the presence of mesangial IgA kidney deposits is still the gold standard for IgAN diagnosis [5]. However, due to the invasive nature of this procedure, its application is not favorable [6, 7]. Despite a huge number of investigations on the IgAN pathogenesis, the underlying mechanisms of this complicated disease are yet to be fully determined [8, 9]. Therefore, discovery of the disease-related pathways and key regulatory agents with a therapeutic/biomarker potential is of utmost necessity to not only shed a light on the disease pathogenicity, but also provide a tool for a non-invasive diagnosis/efficient treatment of IgAN [10].

Today, a wave of researches is dealing with clarifying the role of genes in IgAN pathogenicity. So far, some genes and miRNAs have been identified as key factors in the progression of IgAN [9, 11, 12]. As a matter of fact, due to the complex nature of IgAN, one can assume that no single gene or circuit, but a remarkable number of genes and regulatory networks are involved in the pathogenesis of this disease. Expression profiling with high-throughput techniques has become an extensively applied technology to identify disease associated genes/miRNAs and to identify novel biomarkers in complex diseases like IgAN [10, 13, 14]. In order to translate the produced raw data, they need to be analyzed via various available bioinformatics tools. Moreover, to take the benefits of bigger sample size, researchers could integrate two or more similar datasets in order to achieve more reliable results.

In the IgAN pathogenicity, kidney is thought to be an innocent bystander and the PBMCs as the places for IgA1 production and post-translational modification, are the primary constituents in seeking for the underlying mechanisms of the disease. Accordingly, a huge number of experiments have shown the principal role of PBMCs in IgAN pathogenicity [15, 16]. Moreover, due to the lack of a precise and none-invasive strategy for diagnosis of this disease, identifying potential biomarkers in blood is of great interest [17].

**Fig. 1:**
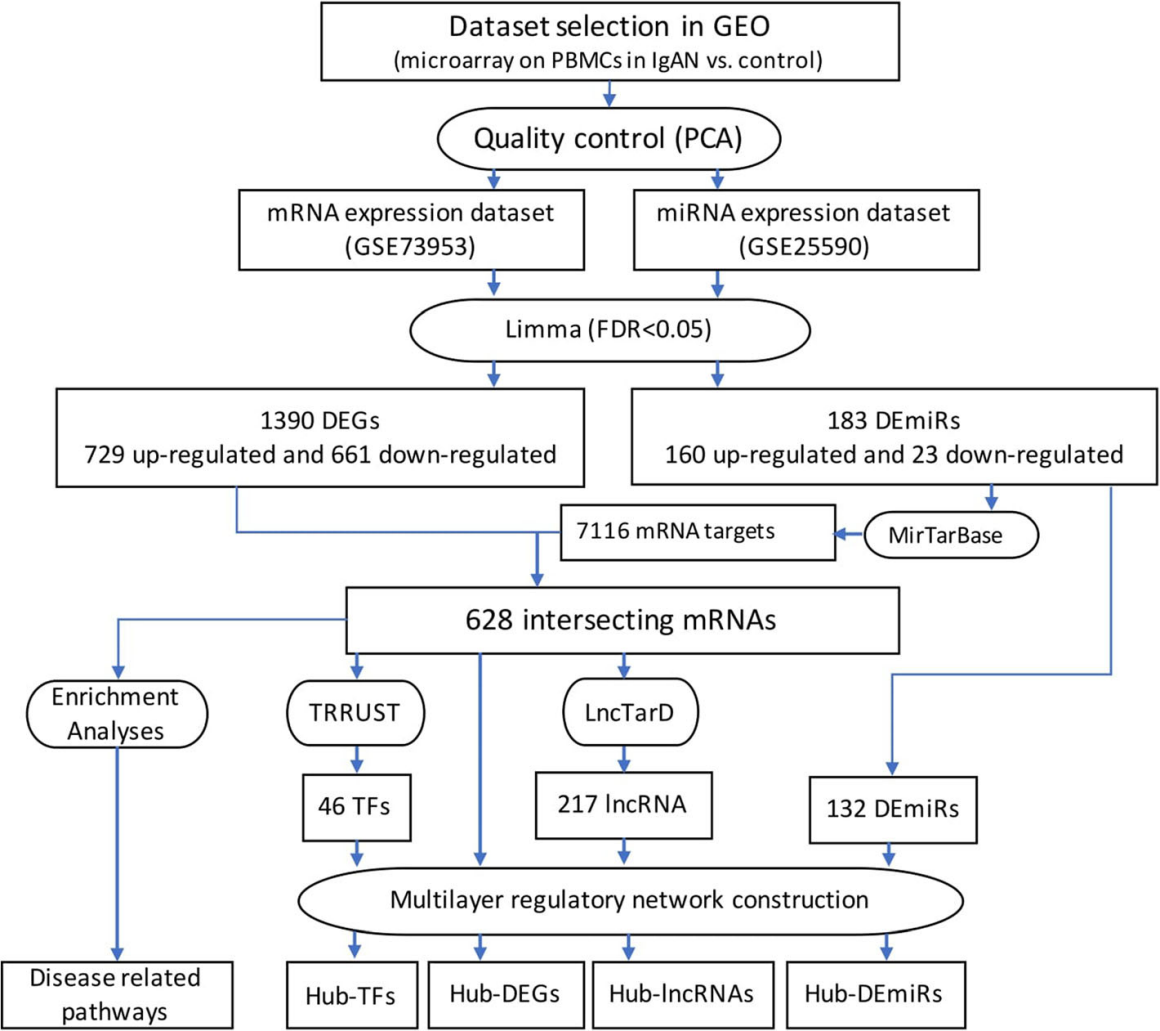
The flowchart representing different steps of the study

The objective of this investigation was to re-analyze and integrate the existing expression profiles coming from peripheral blood mononuclear cells (PBMCs) of IgAN patients. Upon integration of datasets and identifying intersecting mRNAs and different enrichment analyses, a multilayer regulatory network comprising of transcription factors (TFs), miRNAs, and long-non coding RNAs (lncRNAs) will be constructed to catch a holistic view over the interactions and involvement of hub molecules in the PBMCs of IgAN patients. In this study, we tried to identify a specific molecular signature, novel therapeutic targets, potential biomarkers, and core involved pathways in the pathogenesis of IgAN. Different steps of this experiment are demonstrated in Fig. 1.

## Methods

### Dataset selection

Gene Expression Omnibus (GEO) database, as a freely accessible data archive, disseminating functional genomic datasets (https://www.ncbi.nlm.nih.gov/gds) was used for searching and achieving datasets [18]. “IgAN” AND “blood” AND/OR “miRNA” keywords were used in searching for related datasets. Principle component analysis (PCA) using R software (Version 1.2.5033), was used for sample quality control and clustering.

### Identification of differentially expressed miRNAs (DEmiRs) and differentially expressed genes (DEGs)

Identification of DEGs and DEmiRs were performed using NetworkAnalyst website (http://www.networkanalyst.ca). After performing normalization procedures (variance stabilizing normalization followed by quantile normalization), Limma was utilized for identification of DEmiRs and DEGs in the selected datasets. False discovery rate (FDR) < 0.05 cutoff was applied to select the most significant DEmiRs and DEGs. Volcano plots for the DEGs and DEmiRs were built using the R software.

### Identification of intersecting mRNAs

In this step, at first, the DEmiR-target genes were identified using Mienturnet online platform (http://userver.bio.uniroma1.it/apps/,mienturnet/). In this web tool, MirTarBase option was checked to achieve the most reliable validated DEmiR-target genes. After that, a web-based Venn-diagram tool (http://bioinformatics.psb.ugent.be/webtools/Venn/), was applied to identify the intersecting mRNAs between the DEmiR-target genes and the DEGs from the mRNA dataset.

### Enrichment analysis

Gene ontology analysis (molecular function, biological process, and cellular component) was performed using EnrichR, a web-based tool for gene set enrichment analysis)https://amp.pharm.mssm.edu/Enrichr/). Pathway analysis was performed using the same tool based on Reactome database. FunRich (http://www.funrich.org/) was used to represent the enrichment analysis results [19].

### Construction of a multilayer regulatory network and identification of hub nodes

The STRING (version 11) with selecting the highest interaction score (highest confidence: 0.900) was used for the construction of an interactive network among DEGs. TRRUST (version 2.0) database was utilized for the prediction of the DEGs-related transcript factors (TFs) [20, 21].

DEGs and DEmiRs related LncRNAs were selected among all the curated lncRNAs in the LncTarD (www.biocc.hrbmu.edu.cn/LncTarD). After merging and construction of a multilayer network using CytoScape (vesrion: 3.2.0) [22], topmost DEGs, DEmiRs, TFs, and lncRNAs were identified separately using cytoHubba plugin [17] in CytoScape.

## Results

### Dataset selection, extraction, and quality control

Based on our considerations in searching for transcription profiles of IgAN blood samples (PBMCs), two mRNA (GSE58539 and GSE73953) and one miRNA (GSE25590) datasets, were obtained from GEO. The mRNA dataset “GSE73953” included 15 samples from patients with IgAN and two pooled control samples from 16 healthy individuals. The used microarray platform was GPL4133 Agilent-014850 Whole Human Genome Microarray 4x44K G4112F. The miRNA dataset “GSE25590” included 7 samples from healthy individuals and 7 samples from IgAN patients. The samples were assessed using GPL7731 Agilent-019118 Human miRNA Microarray 2.0 G4470B platform. To explore the similarity of the samples in each dataset, sample-level quality control was performed using Principal Component Analysis (PCA). PCA, which is known as a standard technique aiming to reduce the dataset dimensionality, can also be a good tool for representation of the dataset quality [23]. In a so-called good quality dataset, the case and control samples are clustered, separately. Here, due to an inappropriate sample clustering pattern, “GSE58539” dataset was removed from the study and two “GSE73953” and “GSE25590” datasets were selected for further analysis.

**Fig. 2:**
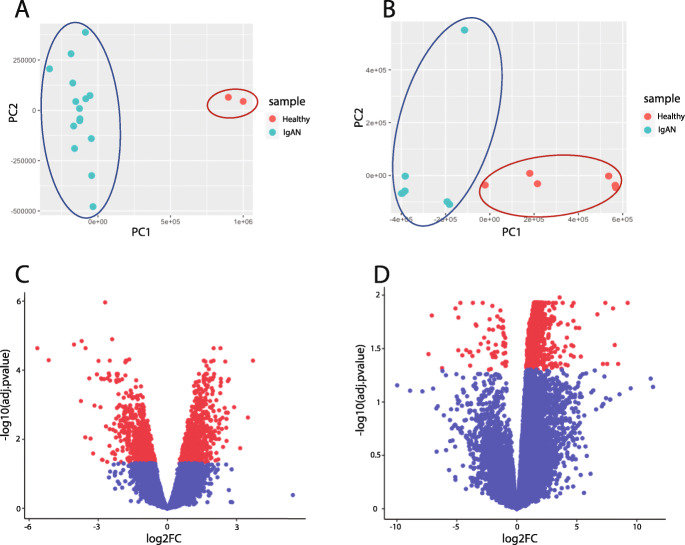
PCA clustering pattern of the healthy and IgAN patient’s samples in mRNA “GSE73953” (**a**) and miRNA “GSE25590” (**b**) datasets. Volcano plots representing the DEGs in the mRNA (**c**), and DEmiRs in the miRNA datasets (**d**)

Another main advantage of PCA clustering is to find possible sample outliers. Removing the sample outliers will give a better sample clustering (case vs. control) also more efficient data analysis. In the case of miRNA data set “GSE25590”, after PCA analysis and removing one healthy sample (as the outlier sample), more significant DEmiRs were identified in this dataset. The other dataset (mRNA dataset; GSE73953) had no outlier and the ‘healthy vs. patients’ samples were separated on PC1 in the PCA plot (Fig. 2a and b).

### 623 Intersecting mRNAs were identified after analysis of mRNA and miRNA datasets

All DEGs and DEmiRs were identified after sample grouping, normalization and analyzing the expression profiles using limma. Considering the FDR, 1390 significantly dysregulated genes (664 down-regulated and 729 up-regulated DEGs), were selected from the analyzed mRNA dataset “GSE73953” (Fig. 2c). In case of DEmiRs, 204 significantly dysregulated miRNAs (149 up-regulated and 55 down-regulated DEmiRs), were identified (Fig. 2d). The topmost differentially expressed mRNAs and miRNAs according to log2FC and adjusted *p*-value are listed in Table 1.

**Table 1:** List of top 20 up- and down-regulated DEGs and DEmiRs in the analyzed datasets

	No	DEGs	Log2FC	Adj.p-value	DEmiRs	Log2FC	Adj.p-value
**Up-regulated**	1	SEC24C	3.709	0.000	miR-146b-3p	8.166	0.029
	2	IFI27	3.492	0.002	miR-493-5p	8.020	0.013
	3	UQCRB	3.156	0.018	miR-516a-3p	7.537	0.044
	4	CARD16	2.893	0.001	miR-668-3p	7.179	0.044
	5	SDHC	2.667	0.000	miR-424-3p	6.713	0.015
	6	CARD17	2.621	0.001	miR-708	5.338	0.037
	7	TSC22D3	2.62	0.000	miR-92a-2-5p	5.201	0.020
	8	TPMT	2.564	0.011	miR-9-5p	4.744	0.037
	9	NOTCH1	2.563	0.002	miR-548d-3p	4.734	0.011
	10	CYP27A1	2.551	0.004	miR-124-3p	4.718	0.017
**Down-regulated**	1	GPR78	−5.642	0.000	miR-935	−7.086	0.015
	2	SLC22A7	−5.147	0.000	miR-920	−6.232	0.048
	3	LRFN1	−4.052	0.000	miR-891a-5p	−4.878	0.032
	4	PRB3	−3.751	0.000	miR-488-3p	−4.870	0.016
	5	SLC26A1	−3.718	0.001	miR-650	−4.703	0.011
	6	SHANK1	−3.561	0.008	miR-137-3p	−4.545	0.036
	7	CACNA1H	−3.550	0.000	miR-372-3p	−3.937	0.034
	8	NLGN2	−3.389	0.000	miR-578	−3.889	0.045
	9	TMEM217	−3.334	0.009	miR-220a	−3.699	0.037
	10	CLSTN1	−3.221	0.025	miR-607	−3.431	0.000

**Fig. 3:**
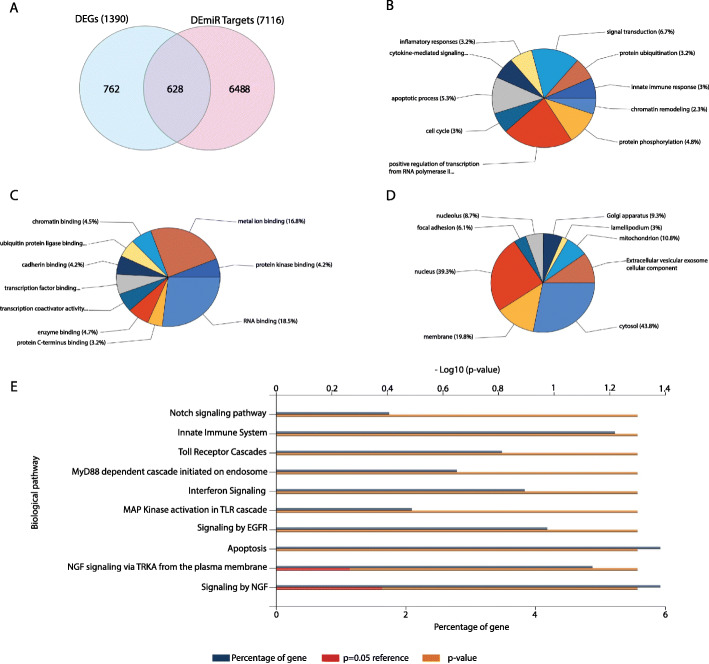
**a**: Venn diagram representing the intersecting mRNAs among the identified DEGs and DEmiR-gene targets. **b**: Topmost enriched biological process, **c**: molecular function, **d**: cellular component GO terms and **e**: Reactome pathways for the intersecting mRNAs

After identification of 7116 DEmiR targets for all the up- and down-regulated DEmiRs, and considering DEGs from the mRNA dataset, 628 intersecting mRNAs were selected as the most reliable differentially expressed mRNAs in the PBMCs of IgAN patients (Fig. 3a). These intersecting mRNAs, then were subjected to further enrichment analyses and network constructions.

### Enrichment analysis revealed the involvement of different pathways in the pathogenesis of IgAN

The topmost enriched pathways and GO terms for all the 628 intersecting mRNAs are shown in Fig. 3b-e. According to the GO enrichment analyses, top enriched biological process GO terms included “Positive regulation of transcription from RNA polymerase II”, “Signal transduction”, “Apoptosis process” and “Protein phosphorylation” (Fig. 3b). Also, “RNA binding”, “Metal ion binding”, “Protein kinase binding” and “Transcription factor binding” were among the most enriched molecular function GO terms (Fig. 3c) for the selected mRNAs. Likewise, the hub DEGs were mainly enriched in “Cytosol” and “Nucleus” and “Membrane” compartments (Fig. 3d). According to the Reactome pathway enrichment analysis, intersecting mRNAs were mainly associated with the “Innate immune system”, “Apoptosis”, “Toll-receptor cascades”, “NGF and EGFR signaling”, “Interferon signaling”, “Notch signaling” as well as “MyD88 dependent cascade initiated on endosome” (Fig. 3e).

### The constructed multilayer regulatory network highlighted the key regulatory elements

**Fig. 4:**
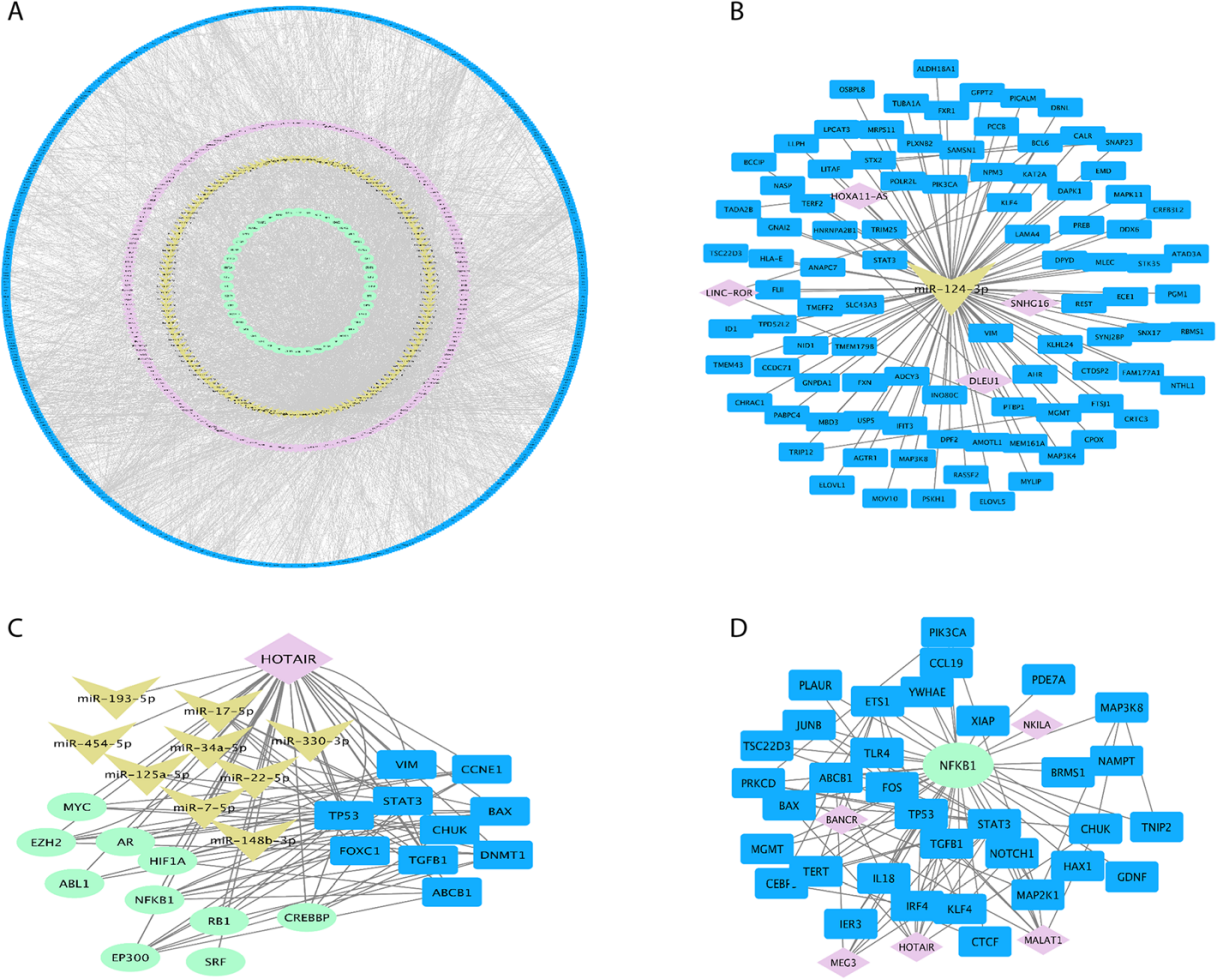
Multilayer regulatory network (**a**), and its derived sub-networks showing top regulatory molecules and their targets (**b-d**). **a**: Multilayer regulatory network comprising of the intersecting mRNAs (DEGs), DEmiRs, and their related TFs and lncRNAs. The constituents of this multilayer network are including 46 transcription factors (green circle), 132 DEmiRs (yellow circle), 217 LncRNAs (magenta circle), and 598 DEGs (blue circle). The DEmiRs and DEGs with no interactions are omitted from the network. **b**: Top miRNA molecule (miR-124) (in case of degree centrality and log2FC in the DEmiR dataset), **c**: top lncRNA (HOTAIR), (D): top transcription factor (NF-κB) and their interactions. *DEGs, DEmiRs, lncRNAs and TFs are colored in blue, yellow, violet and green, respectively. The multilayer regulatory network is accessible at network data exchange (NDEx) server by the below link: [https://public.ndexbio.org/#/network/a1d9669e-7daf-11eb-9e72-0ac135e8bacf?accesskey=c229bf52538f314edd2617ce1f6c6e6cd81ebcbef1437c6d7c81167c3590d973]

To evaluate three levels of regulatory factors affecting the expression of the DEGs (intersecting mRNAs), a multilayer regulatory network including DEGs interactive network and their related TFs, DEmiRs and lncRNAs was constructed and analyzed (Fig. 4a). In a stepwise manner, after the construction of a regulatory network using DEGs and DEmiRs, their related TFs and lncRNAs were identified separately and inserted into the network as other regulatory layers.

In this multilayer network which comprised of 993 nodes and 4407 edges, the topmost nodes from each layer were identified according to degree centrality, independently. Among all DEGs, the top ones, including 30 DEGs with high degree scores in the network were identified as hub-DEGs. Likewise, 3 TFs, 6 miRNAs and 10 lncRNAs were identified based on their scores (degree score) in the multilayer network (Table 2). The selection of hub molecules was based on their degree centrality in the network and 5% of molecules in each group with highest score was introduced as hub molecules.

**Table 2:** List of hub-DEGs, and topmost DEmiRs, TFs and lncRNAs in the constructed multilayer regulatory network

Type	Name	Degree	Type	Name	Degree
**DEGs**	TP53	112	**lncRNAs**	HOTAIR	32
	STAT3	73		MEG3	24
	JUN	46		H19	21
	FOS	43		MALAT1	20
	DNMT1	38		UCA1	17
	NOTCH1	37		CDKN2B-AS1	14
	TGFB1	36		GAS5	10
	E2F3	34		NEAT1	10
	CREB1	34		PVT1	10
	XIAP	34		HULC	9
	SUMO1	33			
	VIM	32	**TFs**	EZH2	74
	ETS1	29		MYC	56
	ABCB1	27		NF-κB	39
	COX6B1	26	**DEmiRs**	hsa-mir-16-5p	134
	TERT	25		hsa-mir-92a-3p	108
	CEBPB	25		hsa-let-7b-5p	105
	CRK	24		hsa-mir-17-5p	102
	TXNIP	24		hsa-mir-124-3p	97
	CCNE1	22		hsa-mir-93-5p	95
	FOXK1	22			
	SNRPD1	21			
	BAX	21			
	TAOK1	21			
	CRCP	21			
	BAX	21			
	TBL1XR1	20			
	HSP90AB1	20			
	Klf4	19			
	HNRNPA2B1	19			

Topmost DEmiRs in the network were comprising of miR-16, miR-92a, let-7a, miR-17, miR-124, and mir-93a. miR-124-3p with log2FC: 4.7 in the DEmiR dataset (a member of the top 10 DEmiRs based on the fold change), could be considered as a non-invasive biomarker for detection of IgAN (Fig. 4b). Likewise, let-7b as another hub-DEmiR was identified as a top DEmiR according to the adjusted *p*-value in the DEmiR dataset.

Due to the importance of gene expression regulation at the transcription level, we were interested to find other regulatory elements like lncRNAs and TFs which could affect the expression of the identified DEGs. LncRNAs are shown to play a remarkable role in transcriptional and post-transcriptional regulations. These molecules also can act as decoys for miRNA binding and form complex regulatory networks that regulate the abundance of miRNA molecules [24]. Among all the curated lncRNAs in the LncTarD database, 217 lncRNA was identified to have interactions with the DEmiRs, DEGs, and the predicted TFs in the regulatory network. In the case of centrality score, HOTAIR was identified as the top lncRNA interacting with 9 DEmiRs, 10 DEGs and 10 TFs in the regulatory network (Fig. 4c). MEG3 and H19 were other top ranked lncRNAs interacting with 24 and 21 DEmiRs/DEGs/TFs in the constructed regulatory network. TFs as other core elements in regulatory machinery also were considered for construction of the multilayer regulatory network. Among all the predicted TFs, NF-κB (nuclear factor-kappa B1) was identified as the top TF interacting with 39 DEGs in the network (Fig. 4d). Other TFs like MYC and EZH2 also were identified as top TFs interacting with both DEGs and lncRNAs.

## Discussion

Re-analyzing and integration of omics-based expression profiles could be an advantageous tactic to catch a holistic view of the involving genes and miRNAs in the IgAN pathogenesis. Until now, several re-analyzes have been performed on the IgAN related expression profiles, mostly on kidney tissue samples of the patients [25–28]. As far as we know, there is just one experiment conducting a meta-analysis study seeking DEGs in the PBMCs of IgAN patients. However, the mentioned experiment is suffering from a lack of dataset quality control checking and not considering other regulatory elements like miRNAs, or lncRNAs in introducing hub-DEGs [29]. In the present study, following quality control checking, we performed re-analysis and integration of two expression datasets (mRNA and miRNA) related to PBMCs of IgAN patients. By applying a systems biology approach, we identified several key genes, TFs, miRNAs, and their related lncRNAs which may have a potential role in the pathogenesis of IgAN. According to the results of pathway enrichment analysis, the intersecting mRNAs were mainly enriched in apoptosis, innate immunity, and NGF signaling pathways.

P53 and XIAP (X-linked inhibitor of apoptosis protein), were among the top DEGs with the highest degree centralities in the regulatory network. These two proteins are considered as the main participants in controlling the apoptosis and proliferation of cells. However, the activity of these two regulatory constituents is unlike; While p53 activity is in the favor of apoptosis and preventing the cell proliferation, XIAP action is to inhibit caspase 3, 7, and 9 and stop the apoptosis [30–32]. Considering the well-known roles of p53 and XIAP in controlling apoptosis [33], these proteins might take a part in controlling the PBMCs proliferation in IgAN patients. Besides, similar to the previous results [34], our findings also pointed to the apoptotic phenotype of PBMCs in IgAN patients. Moreover, based on some evidence, P53 can suppress autoimmunity (e.g., systemic lupus erythematosus) by suppressing T cell activity while the precise mechanisms are still unknown [35].

Other than apoptosis, “innate immunity”, “toll-like receptor cascades” and “interferon signaling” were other enriched pathways for the identified DEGs in the PBMCs of IgAN patients. Such enrichments were predictable due to the autoimmune nature of IgAN disease. Similar to previous findings, the involvement of these pathways could be an indication for hyper-activation and enhancement of antigen processing pathways in PBMCs of IgAN patients [36, 37]. Dysregulation of innate immunity has also been comprehensively suggested in IgAN patients [38].

Toll-like receptors (TLRs) are known as the main components of the innate immune system. The upregulation and higher activity of these receptors in IgAN samples have been shown by previous experiments [39, 40]. Following ligand binding, TLRs trigger several cascades that finally result in the activation of immune system. Through the production of inflammatory cytokines, these receptors have been shown to induce glomerular damages in IgAN patients [41]. Moreover, interaction of some TLRs (e.g., TLR1, TLR2) could result in the activation of Nuclear actor Kappa B Subunit 1 (NF-κB), as well as various immune cells like keratinocytes, dendritic cells, mast cells, B cells, and NK cells [42]. Therefore, these receptors could be considered as potential targets of more investigations regarding the IgAN pathogenesis.

Nerve growth factor (NGF) signaling was another main enriched pathway for the DEGs in PBMCs of IgAN patients. NGF as a prototypical example of neurotrophic factors is mainly involved in the growth, proliferation and differentiation of neurons [43]. However, the role of NGF in the proliferation of immune cells, stimulation of IgM, IgA and IgG production in lymphocytes, as well as regulation of inflammatory mediators like IL-1 beta, TNF-alpha, and IL-6 have been shown previously [44, 45]. Moreover, some findings have revealed the mediatory role of NGF in autoimmune diseases like multiple sclerosis, rheumatoid arthritis, and systemic lupus erythematosus [43]. Other findings also disclosed an increased serum level of NGF, as well as higher NGF receptor expression in PBMCs of glomerulonephritis patients [46]. The findings of the present study also could be a shred of evidence for the contribution of NGF signaling in the pathological process of IgAN disease.

Generally, the involvement of all above-mentioned pathways along with other enriched pathways like notch signaling and EGFR pathways could point to an aberrant proliferation rate and distortion of PBMCs in the IgAN patients. Involvement of the enriched pathways may provide new explanations about the underlying immunopathological mechanisms of IgAN.

In the next step, a multilayer regulatory network was constructed to identify different hub molecules as the main potential participants in the pathogenesis of the disease. The concept of constructing a multilayer network was to achieve a holistic aspect of regulatory interactions in the PBMCs of IgAN patients. Similar experiments in this area of research only consider DEGs to construct an interactive regulatory network, while skipping other regulatory factors like TFs, miRNAs, and lncRNAs. Such studies suffer from a narrow view and ignore the complex regulatory nature of the cell. Accordingly, a multilayer regulatory network is thought to be more realistic and closer to the complex nature of cellular regulatory machinery. The key regulatory constituents in the multilayer network are listed in Table 2. Here, we introduced top DEGs with the highest degree scores in the network as potential biomarker/therapeutic targets. Moreover, in case of TFs, NF-κB was identified the top TFs in regulating the expression of DEGs in IgAN patients. The trace of NF-κB in IgAN pathogenicity has been previously shown, where toll-like receptors trigger a cascade of intracellular messages that finally leads to the activation of this transcription regulator [47]. The consequence of such activation is the cellular release of different cytokines and chemokines, which may participate in aberrant galactosylation of IgA1 [48].

MiRNAs as other main regulatory elements also could play a major role in immunological and pathological aspects of IgAN. Along with their regulatory roles, higher stability in the biological milieu makes these small RNAs as potent non-invasive biomarkers for the early detection of diseases. In case of DEmiRs in the constructed regulatory network, most of the top miRNAs including miR-16, miR-92, miR-17, and let-7b have been shown to play a direct role in controlling the cell cycle and cell proliferation [49–52]. miR-124-3p as another hub-DEmiR in the regulatory network was also among the top 10 DEmiRs in the DEmiR dataset (log2FC: 4.7). According to previous experiments, miR-124 has a crucial role in the innate immunity system and fine-tuning the toll-like receptor responses via targeting signal transducer and activator of transcription 3 (STAT3) [47]. The results of our analysis also pointed to the negative regulatory role of miR-124 in inflammatory responses and introduced this miRNA as a therapeutic target, as well as a non-invasive biomarker for detection of IgAN disease.

Back to the multilayer regulatory network and talking about regulatory roles of miRNAs, let-7b was introduced as another top DEmiR able to target several DEGs and thought to play a pivotal role in the IgAN process/pathogenesis. Up-regulation of this miRNA in PBMCs of IgAN patients and its role in the glycosylation process of IgA with regulation of N-acetylgalactosaminyltransferase 2 (GALNT2) enzyme was previously shown [53, 54]. Overexpression of let-7b in PBMCs of IgAN patients might be a reason for the reduced expression levels of GALNT2 enzyme (log2FC: − 4.43 in the DEG dataset), and consequently aberrant galactosylation of IgA1 in patients. As a result, based on this analysis and previous findings, this miRNA could be considered as a potential therapeutic target for IgAN.

Another layer of the constructed network was specified to lncRNAs as regulatory molecules showing outstanding potential in pre- and post-transcriptional regulations [24]. Despite the regulatory role of lncRNAs, their biomarker potential in different types of kidney diseases like membrane nephropathy [55], diabetic nephropathy [56] and IgAN nephropathy [57] have been proposed by several research teams. In this investigation, after importing different curated lncRNAs in the constructed network, HOTAIR was introduced as the topmost lncRNA in regulating the DEGs and DEmiRs in IgAN pathogenicity.

HOTAIR as an anti-sense lncRNA is involved in epigenetic silencing of various genes by recruiting the PRC2 complex, trimethylation of H3K27 across the HOXD locus and finally repressing the transcription [58]. According to recent findings, transcriptional repression of these RNA molecules in immune cells could lead to NF-κB activation and consequently inflammatory responses [59]. Likewise, the potential role of HOTAIR in provoking inflammation was observed in a study on rheumatoid arthritis patients [60]. Here, we also proposed the probable role of this lncRNA in IgAN disease and introduced this RNA molecule as a key target of further analysis in IgAN disease.

## Conclusion

In conclusion, the results of this survey add a piece of evidence to the possible involvement of some pathways like NGF signaling and toll-like receptor pathways in the pathogenesis of IgAN. The constructed multilayer regulatory network introduced several hub-DEGs (Tp53, STAT3, Jun, etc.), DEmiRs (miR-124, let-7b, etc.), TFs (NF-kB, etc.), and lncRNAs (HOTAIR, etc.) as potential factors in the pathogenesis of IgAN. MicroRNA-124 and HOTAIR as two RNA molecules were introduced as potential non-invasive biomarkers in IgAN disease. All in all, the selected hub-molecules from the constructed multi-layer regulatory network could be reliable choices of further investigations and validations aiming to diagnose, treat and clarify the hidden pathological aspects of IgAN, this enigmatic disorder.

## Data Availability

The datasets analyzed by the current study are available in the GEO repository, [https://www.ncbi.nlm.nih.gov/geo/query/acc.cgi?acc=GSE73953, https://www.ncbi.nlm.nih.gov/geo/query/acc.cgi?acc=GSE25590].
